# PFI1785w: A highly conserved protein associated with pregnancy associated malaria

**DOI:** 10.1371/journal.pone.0187817

**Published:** 2017-11-09

**Authors:** Claire Kamaliddin, Virginie Salnot, Marjorie Leduc, Sem Ezinmegnon, Cédric Broussard, Nadine Fievet, Philippe Deloron, François Guillonneau, Gwladys I. Bertin

**Affiliations:** 1 MERIT, IRD, Université Paris 5, Sorbonne Paris Cité, Paris, France; Laboratoire d’Excellence GR-Ex; DHU Risques et grossesse; Paris, France; 2 3P5 Proteomic facility, COMUE Sorbonne Paris Cité, Université Paris Descartes, Paris, France; 3 Centre d’Etude et de Recherche sur le paludisme associé à la Grossesse et à l’Enfance, Université d’Abomey-Calavi, Cotonou, Benin; Centro de Pesquisas Rene Rachou, BRAZIL

## Abstract

Pregnancy-associated malaria (PAM) is one of the severe forms of *Plasmodium falciparum* infection. The main antigen VAR2CSA is the target of vaccine development. However, the large size of VAR2CSA protein and its high degree of variability limit to the efficiency of the vaccination. Using quantitative mass spectrometry method, we detected and quantified proteotypic peptides from 5 predicted PAM associated proteins. Our results confirmed that PFI1785w is over-expressed in PAM samples. Then, we investigated PFI1785w variability among a set of parasite samples from various endemic areas. PFI1785w appear to be more conserved than VAR2CSA. PFB0115w, another PAM associated protein, seems also associated with the pathology. Further vaccination strategies could integrate other proteins in addition to the major VAR2CSA antigen to improve immune response to vaccination.

## Introduction

During *Plasmodium falciparum* asexual cycle, mature forms reshape the erythrocyte membrane, inducing knobs formation and parasite’s proteins expression within erythrocyte surface [1]. Chondroitin Sulfate A (CSA) expressed in the placenta is the host’s receptor for pregnancy-associated malaria (PAM), a severe form of *Plasmodium falciparum* infection occurring in pregnant women [2]. VAR2CSA, a member of the *Plasmodium falciparum* erythrocyte membrane protein 1 (PfEMP1) variant antigen family, mediates binding to CSA [3]. It has been shown that acquired immunity against VAR2CSA prevent parasite adhesion to placenta, as women become resistant over 1–2 pregnancies as they acquire functional antibodies that block placental adhesion [4,5]. On-going PAM vaccine development is based on recombinant N-terminal part of VAR2CSA protein, or specific domains involved in VAR2CSA-CSA interaction [6,7]. VAR2CSA vaccination may face difficulties due to the variability of VAR2CSA. As VAR2CSA belongs to the PfEMP1 protein family, it is a large protein (350 kD) and highly variable. A study of natural polymorphism of the ID1-DBL2Xb—the leading candidate of the vaccine against PAM in *P*. *falciparum* population–revealed the sequence variation of VAR2CSA in worldwide parasite populations, even though the sequence might be conserved enough for immunization of patients [8]. Actually, antibodies against full-length VAR2CSA inhibit the binding of homologous parasites isolates to a stronger extent than the heterologous ones, even if a cross-reactive antibodies inducing region of VAR2CSA has been revealed [9].

However, other membrane-expressed proteins have been identified in PAM and may interfere with infected erythrocytes adhesion to endothelium, PAM-specific binding protein expression or immunological response to infection. Some specific families of *P*. *falciparum* proteins are described, such as the PHIST proteins. They are known for being implicated in protein trafficking and intercellular communication, and are associated within the host cytoskeleton [10]. The PHIST family is divided into 3 sub-classes (a, b, c). PHISTb protein family is known for being associated with the periphery of host erythrocyte [11]. Other membrane associated protein family, such as RIFIN and STEVOR are studied as potentially involved in parasite’s sequestration [12,13].

Several proteins have been identified as over-expressed in PAM field isolates through transcriptomic studies [14,15], including PFI1785w, identified as associated with PAM in transcriptomic [14,15] and proteomic [16,17] studies. The role of the invariant proteins expressed by placental parasites has recently been reviewed [6], highlighting the presence of PFI1785w and PFB0115w in PAM samples. However, In 2013, a subset of 6 membrane-associated proteins or putative proteins has been shown as preferentially expressed in 10 PAM field isolates compared to 10 uncomplicated malaria (UM) clinical samples using mass spectrometry based proteomic analysis [16]. These proteins were VAR2CSA, PFI1785w, PFF0325c, PFB0115w, PF14_018 and PFA_0410w. The aim of this study was to confirm the level of expression of these proteins, except VAR2CSA (which is already confirmed in numerous studies [3,17–20]), in PAM and UM field samples. As the presence of a protein is the ultimate proof of its expression by an organism, we decided to analyse the level of these proteins with a proteomic approach. mRNA are only a proxy to the level of expression of a given protein, and many events can alternate with the protein expression [21]. We used an unprecedented more accurate and specific method in malaria proteomic research [22]. Using synthetic proteotypic peptides for each targeted protein, we specifically searched, and quantified these peptides in samples. Since PFI1785w was already described as a PAM associated membrane protein, we studied the sequence of this protein and its variability among samples.

## Materials and method

### Field samples protein study

#### Ethic statement

Ethical clearance was obtained from the Institutional Ethics Committee of the Faculty of Health Science, Abomey-Calavi University (CER-ISBA). Written informed consent was obtained from patients, their parents, or guardians. Research protocol did not interfere with patients care and treatment was given following Benin Ministry of Health guidelines. Ethic approval CER-ISBA (n°39, 16/05/2014 and 17/06/2015) and CER-ISBA (n°90 06/06/2016).

#### Subjects enrolment and samples collection

Samples were collected between September 2015 and September 2016. Inclusion criteria were a positive rapid diagnosis test for HRP-2 confirmed with Giemsa-stained thick blood smear. Placental blood samples were collected at CHU-MEL maternity hospital (Cotonou, Benin) and Centre Medical Saint Joseph (Sô-Ava, Benin) from delivering women. Uncomplicated malaria samples were collected in Centre Medical Saint Joseph from children (< 10 years old) presenting with fever > 37.8°C and no sign of severe malaria. Peripheral blood samples (5 mL) were collected on EDTA anticoagulant.

Two reference strains were used, an FCR3 strain selected for CSA binding, and a non-selected Hb3 strain.

#### Mature parasite stages enrichment

Placental and peripheral blood samples were processed as described [16]. Laboratory strains were cultured in RPMI medium with human serum under (2% O_2_, 5.5% CO_2_, 92.5% N_2_) condition until parasites present trophozoite stages. Mature parasites have been immediately frozen (-80°C) for protein preservation.

#### Proteotypic peptides

Synthetic proteotypic peptides (Table 1) have been designed using identification data from a previous study [16]. Peptides were selected within those allowing protein identification in the majority of samples, and avoiding technical pitfalls such as trypsin mis-cleavage. Peptides were aligned against all NCBI using BLAST® (blastp suite) to ensure their specificity to the protein, and were ordered in solution in AQUA Ultimate quality (ThermoFisher Scientific, San Jose, CA). Each peptide was designed to quantify a given protein.

**Table 1:** Description of the proteins of interest.

	PFI1785w	PF14-0018	PFA-0410w	PFF0325c	PFB0115w
ID PlasmoDB	PF3D7_0936900.1	PF3D7_1401600.1	PF3D7_0108300.1	PF3D7_0606600.1	PF3D7_0202400.1
Accession number (NCBI)	164419780	258597612	124505801	296004626	124800702
Length (Amino acid)	363	478	2221	2414	1192
Proteotypic peptide	SSIGDLIQVIK	SSTLSQGNYSNFK	GHTVDSELTTEDPVIQKK	SIFNEHDNR	LSGNNEQQNNSIPK
Position	168–178	147–159	1212–1229	1642–1650	148–161
Transmembran domain (number)	1	1	0	1	1
Isoelectric Point	6.52	6.53	5.38	4.82	4.47
Molecular Weight (Da)	43055	55826	254991	280360	141848

All five peptides were mixed in equimolar concentration, and 2-fold dilution were performed using a digest from non-infected red blood cells, so that calibration range and samples would be analysed in the closest possible peptidic environment. Dilution ranged from 416 nM to 0.203 nM.

#### Sample preparation for LC-MS/MS analysis

Protein extraction from mature parasite stage isolates was performed with protease inhibitor cocktail set I (Calbiochem, #53913, Merck Millipore). Briefly, whole lysate was solubilized with 1% Triton X-100, then centrifuged at 13,000 rpm for 20 min at 4°C. Pellets were re-suspended in 2% SDS. Protein dosage was performed using BCA protein assay (Pierce #23250). Digested peptides were prepared as described [16].

Thirty-five μg of each digested protein sample was cleaned-up using 6 layers Empore C18 extraction disks (3M) stage tip chromatography for salt removal [23]. Elution occurred in acetonitrile 80% and acetic acid 0.5%. Eluates were concentrated on *speed-vac* concentrator until solvent’s full evaporation before solubilisation in acetonitrile 10% and trifluoroacetic acid 0.1%. Peptides were separated on a C18 RP analytical column (2 μm particle size, 100 Å pore size, 75 μm insider diameter; 25 cm length) with a 157 minutes gradient for MS/MS analysis.

#### Mass spectrometry method

We optimized a targeted single ion monitoring method coupled with a data dependent ms2 (t-sim dd ms2) with a specific inclusion list in the mass spectrometry method. Inclusion list parameters were determined by proteotypic peptides MS analysis. Retention times and m/z were collected. We then selected the most abundant ionization state for each peptide to set up the method. Reproductivity of retention time has been evaluated. Qexactive+ acquisition chain collected data throughout the run.

#### Mass spectrometry data analysis

Raw files from Qexactive+ mass spectrometry were analysed using Skyline software (64 bit 3.6.0.10162, University of Washington, MacCoss Lab, Department of Genome Sciences, UW, available at skyline.gs.washington.edu). Reference sequences for each targeted protein were included in the protein list. *In silico* digestion was performed.

**Fig 1:**
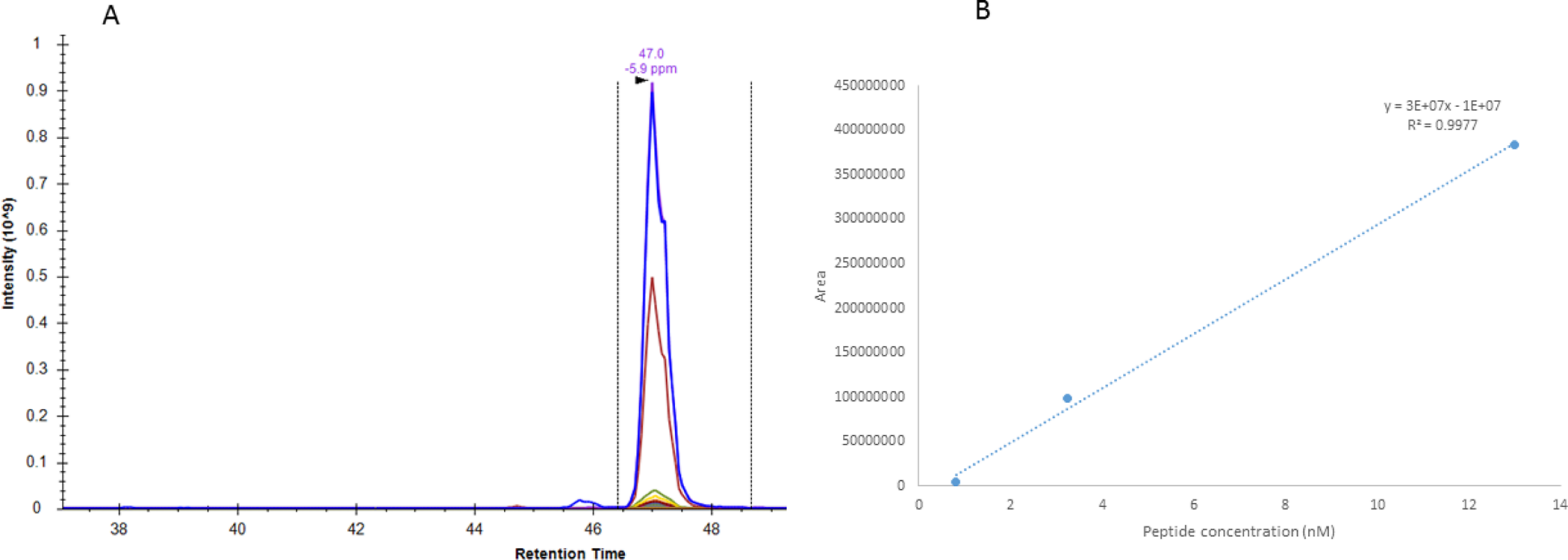
Skyline output and calibration range for GHTVDSELTTEDPVIQKK. Retention time is displayed on x axis and intensity for each ion or fragment is displayed on y axis. Each parent ion and fragment is shown in a different colour. AUC corresponds to the integration of signal under the curve (A). Regression line is obtained using AUC from each concentration point.

Transition ion file was exported to.csv file for quantitative analysis of data. We obtained calibration curve using AQUA peptides fold dilutions and Area Under Curve (AUC) (Fig 1).

#### Data analysis

Concentrations obtained for each peptide in each sample were plotted to compare PAM and UM groups using “R” 3.3.2 software. One-sided Wilcoxon test has been performed to compare peptides concentration in field samples. We considered a test significant for *p <* 0.05.

### Bio-informatics study of *PFI1785w* in genomic data set and predictive protein structure of PFI1785w

#### PFI1785w sequence diversity in P. falciparum laboratory strains

We aligned the reference 3D7 PFI1785w protein sequence against the sequence equivalent for seven other *Plasmodium falciparum* strains (IGH-CR14 (KNG78441.1), MaliPS096_E11 (ETW49354.1), Palo Alto/Uganda (ETW56861.1); UGT5.1 (EWC76603.1), FCH/4 (ETW30803.1), Tanzania (2000708) (ETW36589.1) and Dd2 (KOB87687.1)).

#### Variability in PFI1785w genomic sequence in P. falciparum isolates and predictive consequences on protein sequence

We used 31 genome sets from the Pf3k project, available on https://www.malariagen.net/projects/pf3k, and two genomes from a home-based sequencing study. We selected samples from various area in Asia and Africa. Bam files were downloaded from MalariaGen FTP and aligned against reference 3D7 genome available on PlasmoDB (released 06/26/2017 PlasmoDB-33, http://plasmodb.org/common/downloads/ Current_Release/Pfalciparum3D7/fasta/data/). Sequence variation on chromosome 9 (+) in position 1,462,400 to 1,463,659, corresponding to *PFI1785w* genomic sequence were collected for each sample.

To study the impact of the collected mutation compared to the reference sequence, we removed the intronic sequence and performed an *in silico* translation using Expasy online translate tool (http://web.expasy.org/translate/).

The obtained protein sequences have been aligned using MultiAlin (available at http://multalin.toulouse.inra.fr/multalin/) [24].

#### Predictive protein structure of PFI1785w

We used the Phyre^2^ V2.0 online tool (available at http://www.sbg.bio.ic.ac.uk/phyre2/html/page.cgi?id=index) [25] for analyse of PFI1785w protein sequence.

## Results

### Field samples protein study and mass spectrometry quantification

#### Patients included

Five placental blood samples were successfully processed, as well as 8 UM samples. Average placental parasitemia was 6 455 p/μL (min 1608 p/μL, max 23640 p/μL), while UM average parasitemia was 35 799 p/μL (min 127 p/μL, max 81 740 p/μL). UM samples were mingled in 3 pools, each containing 100μg of proteins. Laboratory strains FCR3-CSA and Hb3 were respectively added to the PAM and UM groups.

#### Proteotypic peptides fold dilutions and calibration range

Proper calibration curve was obtained for 4 out of the 5 studied peptides. Linear regression displayed concentrations and the AUC corresponding to quantity of the detected ion, as displayed on Fig 1. We obtained acceptable linear regression equation for all peptides except LSGNNEQQNNSIPK. R^2^ were from 0.822 to 0.998 (Table 2)

**Table 2:** Linear regression line obtained with Skyline integration of raw files.

Protein	Peptide	Linear regression line	R^2^
PF14_0018	YNVDNSFSEK	AUC = 1242861.48 C + 3566433.00	0.822
PFA_0410w	GHTVDSELTTEDPVIQKK	AUC = 30620627.223 C—11680528.667	0.998
PFI1785w	SSIGDLIQVIK	AUC = 1359079.03 C + 11573121.33	0.966
PFF0325c	SIFNEHDNR	AUC = 4215152.498 C + 180360228.167	0.985
PFB0115w	LSGNNEQQNNSIPK	na	na

Median peptide concentration obtained within the calibration range is displayed for PAM samples (n = 6) and UM samples (n = 4). For PFB0115w (LSGNNEQQNNSIPK), no regression line could be obtained.

#### Peptide detection and concentration

**Fig 2:**
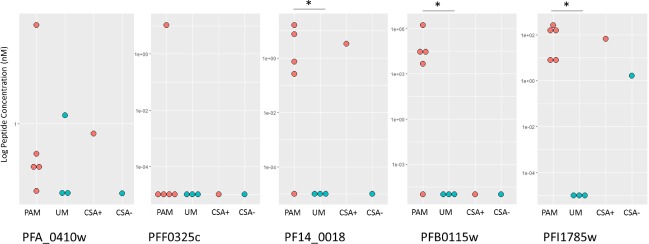
Peptide concentration measured within calibration range of each peptide. Peptide concentrations calculated by linear regression for each studied protein, y axis presents peptide concentration. Each dot represents one sample, and reference strains are displayed separately on the right of each plot (CSA+: CSA adhesion selected parasite strain, CSA-: non selected parasite strain). PAM samples and CSA+ parasite strains display a placental malaria like binding phenotype (in red), and UM samples and CSA- parasite strain display a uncomplicated malaria phenotype (in blue). PF14_0018, PFA_0410w, PFB0115w and PFI1785w are preferentially found in PAM like samples.

Using the calibration range obtained with proteotypic peptides, we were able to calculate mean concentrations in the two clinical sample groups: PAM and UM. Average concentration was considerably higher in PAM samples for SSIGDLIQVIK, the proteotypic peptide for PFI1785w protein. Using Wilcoxon test, we observed statistically significant difference for proteotypic peptide for PFI1785w (*p* = 0.016), PFB0115w (*p* = 0.016) and PF14_0018 (*p =* 0.040). We also observed a preferential expression in PAM samples for PF14_0018, PFA_0410w, but not significant. (Fig 2).

### Bio-informatics study of *PFI1785w* in genomic data set and predictive protein structure of PFI1785w

#### PFI1785w protein sequence in *P*. *falciparum* laboratory strains

Predicted sequences from eight *P*. *falciparum* laboratory strains show a sequence mostly conserved. Protein sequence appeared to be truncated in position 1–60, 129–159 and 278 depending on strains.

#### Variation of PFI785w gene in *P*. *falciparum* isolates

**Fig 3:**
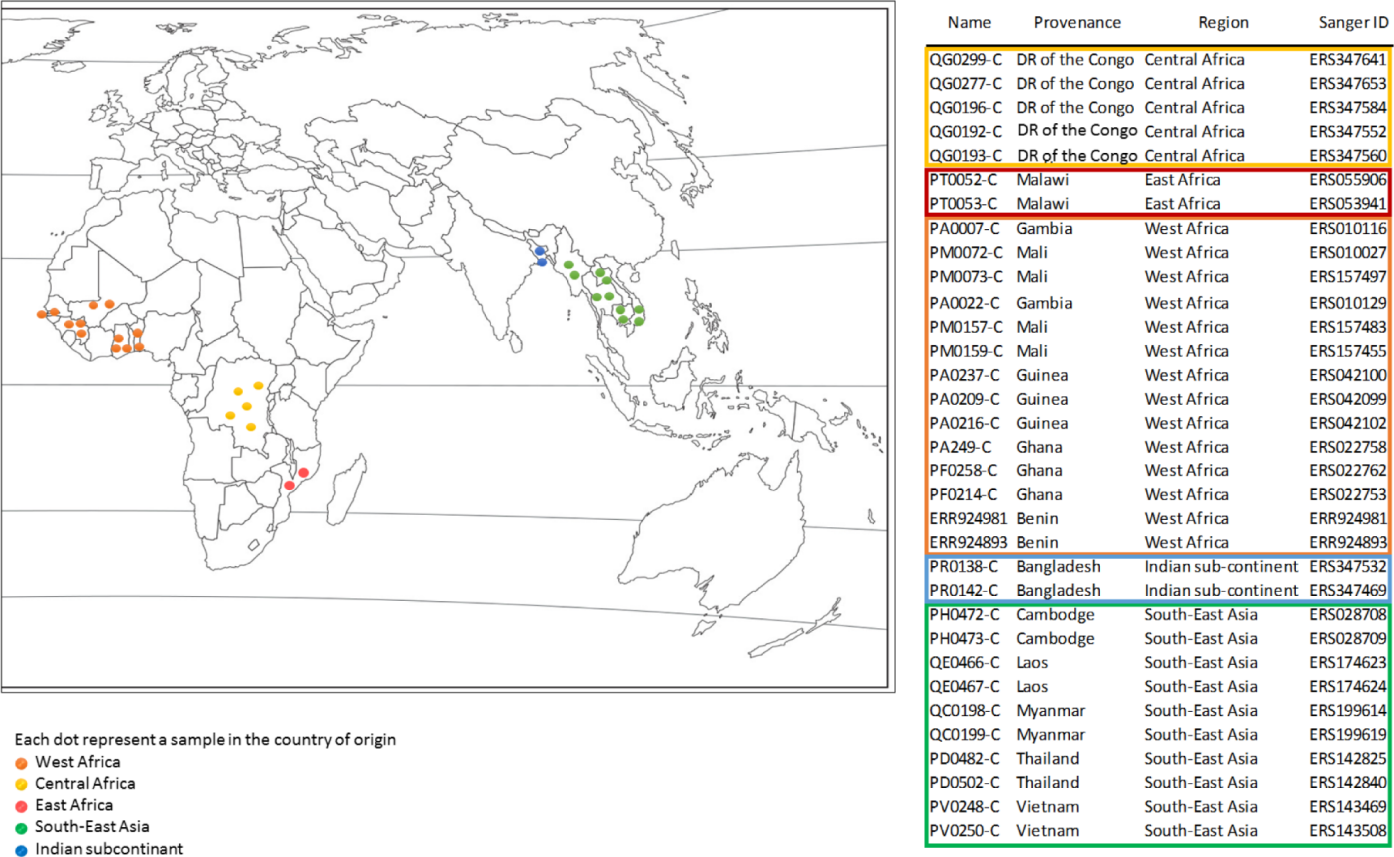
Geographic origin of the samples used for genomic studies of *PFI1785w*.

We selected the genomic data and studied the sequence of *PFI1785w* from 33 field isolates. Fifteen (45%) were from Asia and eighteen (55%) from Africa. Samples information’s are displayed on Fig 3.

**Fig 4:**
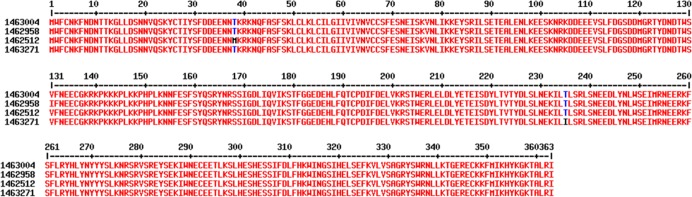
Alignment of PFI1785w sequence with mutation in *pfi1785w* observed in field isolates. Alignment of PFI1785w protein sequences using the full length PFI1785w as a reference (1463004). Sequence names correspond to the position of the mutation found in genomic sequence. Mutation on *PFI1785w* gene (1,462,512 C->T) (12/33 sequences) and (1,463,271 C->T) (1/33 sequences) lead to amino acid changes in protein sequence on position 38 (Thr->Met) and 235 (Thr->Ile).

Eleven sites of mutation were observed. Six of these sites were nucleotide substitution in intron. Five were substitution in exon. One substitution was constant in all samples (T->A in position 1,463,004) and allows the traduction of the entire protein. Other mutations were observed in addition of the 1,463,004 position, leading to 2 amino acid substitutions in final protein (1,462,512 C->T) (12/33 sequences), and (1,463,271 C->T) (1/33 sequences). First amino acid change was on position 38 (Thr->Met), and the second on position 235 (Thr->Ile). Obtained sequences are displayed aligned to the reference sequence with T->A in mutation in position 1,463,004 only (Fig 4).

#### PFI1785w predicted protein structure

**Fig 5:**
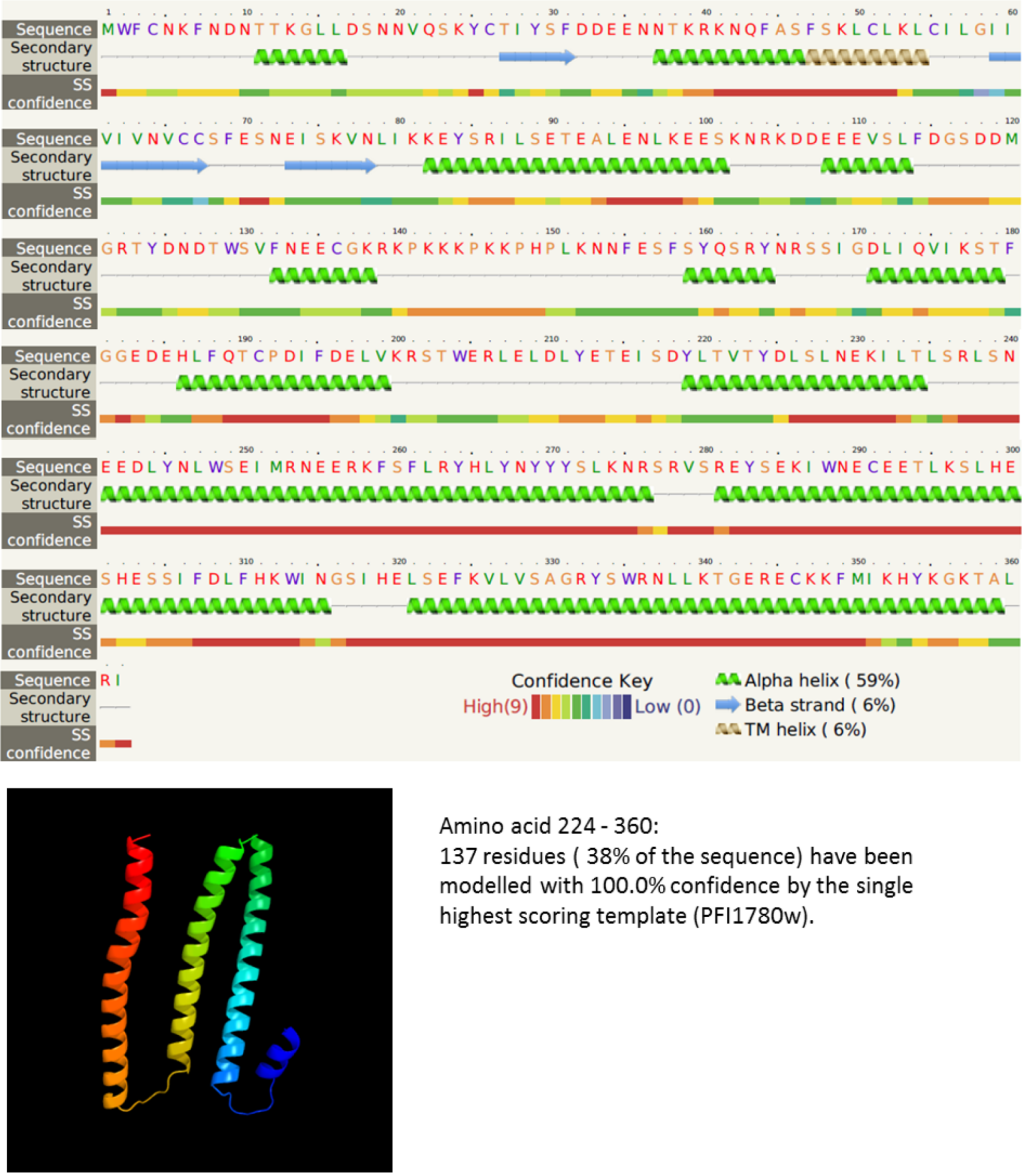
Secondary structure of PFI1785w predicted using Phyre 2 and predictive 3D structure of amino-acid 224–360.

Phyre^2^ tool was used for prediction of the protein secondary structure of the full-length PFI1785w protein sequence. We confirmed the existence of one transmembrane domain from amino acid position 47 to 67. N-terminal segment appeared to be intra-cytoplasmic while C-ter segment is exposed at the erythrocyte surface. Secondary structure prediction on full length protein displayed mostly alpha helices (59% of the structure) alternated with beta strand (6%) and disordered sequences (31%). Transmembrane domain is composed by an alpha helices (Fig 5). The proteotypic peptide used for mass spectrometry analysis is located on the exposed segment of the protein, ensuring a good solubility.

3D model was compromised by the absence of a homologous protein in PDB data set. However, an alignment with excellent confidence (100%) to the phist domain of PFI1780w displayed a proper structure composed of alpha-helix for amino acid 224–360 (extracellular domain) (Fig 5).

## Discussion

From our knowledge, this is the first reported use of single ion target MS approach to quantify malaria surface antigens. We used synthetic peptide fold dilution series to quantify peptides in 5 PAM and 8 UM samples, in addition of CSA-adherent and non-selected *P*. *falciparum* laboratory strains. Those peptides were chosen as a signature from proteins previously identified as preferentially expressed in PAM samples [16]. We confirmed the over expression of PFI1785w in our set of samples (*p* = 0.016). Our quantitative analysis also shows a significantly higher expression of PF14_0018, and PFB0115w PFA_0410w in PAM samples compared to UM samples.

A STRING [26] network analysis of these proteins displays text-mining evidence but co-expression is known only for PFF0325c, PFA_0410w, and PF14_0018. No functional analysis has been reported on this set of proteins. PFB0115w is described for catalytic and transporter activities, and is involved in proteins that traffic within the erythrocyte by alternate export pathways [16]. These proteins might be involved in PAM pathogenicity but their role in adhesion proteins expression or parasite’s adhesion to placenta is yet unknown. However, PFI1785w and PFB0115w might be promising for vaccine development strategies as they were found as associated with PAM in respectively four and three independent studies [14–17], and confirmed with our innovative approach.

The structure of PFI1785 remains predictive. However, PFI1785w belongs to the Poly-Helical Interspersed Sub-Telomeric (PHIST) protein family. PFI1785w is associated to PHISTb sub-class. We performed a 3D modellisation of PFI1785w structure, but we could not predict its 3D conformation due to the lack of template sequences in current database, except for the residues 224 to 360. For those amino acids, the reference protein for structure prediction was PFI1780w, another PHIST protein which has been described as associated with VAR2CSA [27].

From immunological perspective, it has been shown that sera from individuals living in malaria endemic areas recognized recombinant PFI1785w protein, with a higher antibody level in women compared to men [14], suggesting that women encounter the variant more frequently. These precedent results, in addition with our findings, strongly suggest that PFI1785w is involved in PAM, even though the mechanism is yet unknown.

We then investigated the variability of PFI1785w protein sequence among samples. We aligned sequences from eight *P*. *falciparum* strains and did not observe any polymorphism. However, field isolates sequencing data reflect better the natural diversity. Using MalariaGen Pf3k project data and two samples from a home study, we aligned 33 samples from Africa and Asia with the 3D7 reference genome. The samples were chosen to reflect the geographical diversity of *P*. *falciparum* genomes, but were not associated with any clinical status. We confirmed the mutation in position 14360004 A->T already described [14]. Without the mutation, the protein is truncated. This nucleotide substitution was constant among genomes, suggesting that the reference sequence should not be considered for this position, as suggested by Tuikue Ndam and *al* [14]. Apart from this mutation, the sequence remains mostly conserved. Mutation in introns do not affect protein sequences. We observed two predictive protein sequence variations in the 33 tested samples. The first one Thr38Met, was observed in a single sample from Myanmar. As this mutation is located in the intracellular part of PFI1785w, it should not interfere with the immune system recognition of the protein. However, intracytoplasmic mutation might affect protein addressing to membrane or interaction with other surface associated proteins.

The other mutation observed was on amino acid position 235 and therefore is exposed at the erythrocyte surface. As the mutation was observed in 12/33 samples, with no correlation with geographic area, it might represent the natural diversity of the protein. These finding should be investigated in further studies.

No biological data is available to date for the 5 other proteins immunogenicity and structure. *P*. *falciparum* knobs formation occurs during parasite development and involves several mechanisms in protein expression to cell surface. RIFIN, STEVOR, PfEMP-1 and PHIST proteins undergo a specific addressing to the erythrocyte membrane through a peptidic recognition signal (PEXEL motif) [1]. PFI1785w and PF14_0018 display a PEXEL motif. No specific signal has been shown for PFB0115w, PFF0325c and PFA_0410w.

VAR2CSA is the targeted protein for malaria vaccine trials but its large size and sequence variability, as for other *Pf*EMP1 proteins, are obstacles to vaccine development [6]. Our data confirm the involvement of PFI1785w in PAM, and strongly suggest an involvement of PF14_0018, PFA_0410w, and PFB0115w. Structural Studies should be set up to investigate whether these proteins are immunogenic, and if the induced antibody are protective against *P*. *falciparum* infection of the pregnant woman and parasite sequestration in placenta. Up to date, the only study using antibody against recombinant PFI1785w did not show reaction with the surface of life iE [17]. The authors suggest that this is linked to the 3D conformation of the protein, as the recombinant PFI1785w used in their study was a non-conformational epitope. When used with membrane fraction of placental iE, the PFI1785w antisera reacted with a protein of the predicted size, suggestion the membrane associated localisation of PFI1785w.

Virus Like Particle (VLP) based vaccine allows to combine antigens for immunization [7,28,29]. Using alternative proteins in combination with VAR2CSA could allow an integrative approach in PAM vaccination trial. VLP particles could contain full-length proteins in conformation closer to reality than recombinant protein based vaccines. PFI1785w is smaller VAR2CSA, as the other identified proteins from this study [6], and less variant. Therefore, a combined vaccine with several antigens could diversify the host immune response to pathogen, and ensure a better protection against PAM. Even since our results are still preliminary, they encourage to pursue research on PAM associated membrane proteins from *P*. *falciparum*.

## References

[1] Maier AG, Cooke BM, Cowman AF, Tilley L. Malaria parasite proteins that remodel the host erythrocyte. Nat Rev Microbiol. 2009;7: 341–354. doi: 10.1038/nrmicro2110 19369950 10.1038/nrmicro2110

[2] Andrews KT, Lanzer M. Maternal malaria: Plasmodium falciparum sequestration in the placenta. Parasitol Res. 2002;88: 715–723. doi: 10.1007/s00436-002-0624-5 12122428 10.1007/s00436-002-0624-5

[3] Ayres Pereira M, Mandel Clausen T, Pehrson C, Mao Y, Resende M, Daugaard M, et al Placental Sequestration of Plasmodium falciparum Malaria Parasites Is Mediated by the Interaction Between VAR2CSA and Chondroitin Sulfate A on Syndecan-1. PLoS Pathog. 2016;12 doi: 10.1371/journal.ppat.1005831 27556547 10.1371/journal.ppat.1005831 PMC4996535

[4] Gamain B, Smith JD, Viebig NK, Gysin J, Scherf A. Pregnancy-associated malaria: Parasite binding, natural immunity and vaccine development. Int J Parasitol. 2007;37: 273–283. doi: 10.1016/j.ijpara.2006.11.011 17224156 10.1016/j.ijpara.2006.11.011

[5] Fried M, Nosten F, Brockman A, Brabin BJ, Duffy PE. Maternal antibodies block malaria. Nature. 1998;395: 851–852. doi: 10.1038/27570 9804416 10.1038/27570

[6] Fried M, Duffy PE. Designing a VAR2CSA-based vaccine to prevent placental malaria. Vaccine. 2015;33: 7483–7488. doi: 10.1016/j.vaccine.2015.10.011 26469717 10.1016/j.vaccine.2015.10.011 PMC5077158

[7] Thrane S, Janitzek CM, Agerbæk MØ, Ditlev SB, Resende M, Nielsen MA, et al A Novel Virus-Like Particle Based Vaccine Platform Displaying the Placental Malaria Antigen VAR2CSA. PloS One. 2015;10: e0143071 doi: 10.1371/journal.pone.0143071 26599509 10.1371/journal.pone.0143071 PMC4657905

[8] Bordbar B, Tuikue Ndam N, Renard E, Jafari-Guemouri S, Tavul L, Jennison C, et al Genetic diversity of VAR2CSA ID1-DBL2Xb in worldwide Plasmodium falciparum populations: impact on vaccine design for placental malaria. Infect Genet Evol J Mol Epidemiol Evol Genet Infect Dis. 2014;25: 81–92. doi: 10.1016/j.meegid.2014.04.010 24768682 10.1016/j.meegid.2014.04.010

[9] Bigey P, Gnidehou S, Doritchamou J, Quiviger M, Viwami F, Couturier A, et al The NTS-DBL2X region of VAR2CSA induces cross-reactive antibodies that inhibit adhesion of several Plasmodium falciparum isolates to chondroitin sulfate A. J Infect Dis. 2011;204: 1125–1133. doi: 10.1093/infdis/jir499 21881129 10.1093/infdis/jir499

[10] Warncke JD, Vakonakis I, Beck H-P. Plasmodium Helical Interspersed Subtelomeric (PHIST) Proteins, at the Center of Host Cell Remodeling. Microbiol Mol Biol Rev. 2016;80: 905–927. doi: 10.1128/MMBR.00014-16 27582258 10.1128/MMBR.00014-16 PMC5116875

[11] Tarr SJ, Moon RW, Hardege I, Osborne AR. A conserved domain targets exported PHISTb family proteins to the periphery of Plasmodium infected erythrocytes. Mol Biochem Parasitol. 2014;196: 29–40. doi: 10.1016/j.molbiopara.2014.07.011 25106850 10.1016/j.molbiopara.2014.07.011 PMC4165601

[12] Goel S, Palmkvist M, Moll K, Joannin N, Lara P, Akhouri RR, et al RIFINs are adhesins implicated in severe Plasmodium falciparum malaria. Nat Med. 2015;21: 314–317. doi: 10.1038/nm.3812 25751816 10.1038/nm.3812

[13] Niang M, Bei AK, Madnani KG, Pelly S, Dankwa S, Kanjee U, et al STEVOR is a Plasmodium falciparum erythrocyte binding protein that mediates merozoite invasion and rosetting. Cell Host Microbe. 2014;16: 81–93. doi: 10.1016/j.chom.2014.06.004 25011110 10.1016/j.chom.2014.06.004 PMC4382205

[14] Tuikue Ndam N, Bischoff E, Proux C, Lavstsen T, Salanti A, Guitard J, et al Plasmodium falciparum transcriptome analysis reveals pregnancy malaria associated gene expression. PloS One. 2008;3: e1855 doi: 10.1371/journal.pone.0001855 18365010 10.1371/journal.pone.0001855 PMC2267001

[15] Francis SE, Malkov VA, Oleinikov AV, Rossnagle E, Wendler JP, Mutabingwa TK, et al Six Genes Are Preferentially Transcribed by the Circulating and Sequestered Forms of Plasmodium falciparum Parasites That Infect Pregnant Women. Infect Immun. 2007;75: 4838–4850. doi: 10.1128/IAI.00635-07 17698567 10.1128/IAI.00635-07 PMC2044550

[16] Bertin GI, Sabbagh A, Guillonneau F, Jafari-Guemouri S, Ezinmegnon S, Federici C, et al Differential protein expression profiles between Plasmodium falciparum parasites isolated from subjects presenting with pregnancy-associated malaria and uncomplicated malaria in Benin. J Infect Dis. 2013;208: 1987–1997. doi: 10.1093/infdis/jit377 23901091 10.1093/infdis/jit377

[17] Fried M, Hixson KK, Anderson L, Ogata Y, Mutabingwa TK, Duffy PE. The distinct proteome of placental malaria parasites. Mol Biochem Parasitol. 2007;155: 57–65. doi: 10.1016/j.molbiopara.2007.05.010 17618698 10.1016/j.molbiopara.2007.05.010

[18] Nielsen MA, Resende M, Alifrangis M, Turner L, Hviid L, Theander TG, et al Plasmodium falciparum: VAR2CSA expressed during pregnancy-associated malaria is partially resistant to proteolytic cleavage by trypsin. Exp Parasitol. 2007;117: 1–8. doi: 10.1016/j.exppara.2007.03.002 17442305 10.1016/j.exppara.2007.03.002

[19] Tuikue Ndam NG, Salanti A, Bertin G, Dahlbäck M, Fievet N, Turner L, et al High level of var2csa transcription by Plasmodium falciparum isolated from the placenta. J Infect Dis. 2005;192: 331–335. doi: 10.1086/430933 15962229 10.1086/430933

[20] Salanti A, Dahlbäck M, Turner L, Nielsen MA, Barfod L, Magistrado P, et al Evidence for the involvement of VAR2CSA in pregnancy-associated malaria. J Exp Med. 2004;200: 1197–1203. doi: 10.1084/jem.20041579 15520249 10.1084/jem.20041579 PMC2211857

[21] Vogel C, Marcotte EM. Insights into the regulation of protein abundance from proteomic and transcriptomic analyses. Nat Rev Genet. 2012;13: 227–232. doi: 10.1038/nrg3185 22411467 10.1038/nrg3185 PMC3654667

[22] Gerber SA, Rush J, Stemman O, Kirschner MW, Gygi SP. Absolute quantification of proteins and phosphoproteins from cell lysates by tandem MS. Proc Natl Acad Sci U S A. 2003;100: 6940–6945. doi: 10.1073/pnas.0832254100 12771378 10.1073/pnas.0832254100 PMC165809

[23] Rappsilber J, Ishihama Y, Mann M. Stop and go extraction tips for matrix-assisted laser desorption/ionization, nanoelectrospray, and LC/MS sample pretreatment in proteomics. Anal Chem. 2003;75: 663–670. 12585499 10.1021/ac026117i

[24] Corpet F. Multiple sequence alignment with hierarchical clustering. Nucleic Acids Res. 1988;16: 10881–10890. 2849754 10.1093/nar/16.22.10881 PMC338945

[25] Kelley LA, Mezulis S, Yates CM, Wass MN, Sternberg MJE. The Phyre2 web portal for protein modeling, prediction and analysis. Nat Protoc. 2015;10: 845–858. doi: 10.1038/nprot.2015.053 25950237 10.1038/nprot.2015.053 PMC5298202

[26] Szklarczyk D, Morris JH, Cook H, Kuhn M, Wyder S, Simonovic M, et al The STRING database in 2017: quality-controlled protein-protein association networks, made broadly accessible. Nucleic Acids Res. 2017;45: D362–D368. doi: 10.1093/nar/gkw937 27924014 10.1093/nar/gkw937 PMC5210637

[27] Mayer C, Slater L, Erat MC, Konrat R, Vakonakis I. Structural analysis of the plasmodium falciparum Erythrocyte membrane protein 1 (PfEMP1) intracellular domain reveals a conserved interaction epitope. J Biol Chem. 2012; jbc.M111.330779. doi: 10.1074/jbc.M111.330779 22249178 10.1074/jbc.M111.330779 PMC3293552

[28] Pitoiset F, Vazquez T, Bellier B. Enveloped virus-like particle platforms: vaccines of the future? Expert Rev Vaccines. 2015;14: 913–915. doi: 10.1586/14760584.2015.1046440 25968245 10.1586/14760584.2015.1046440

[29] Jennings GT, Bachmann MF. The coming of age of virus-like particle vaccines. Biol Chem. 2008;389: 521–536. 18953718 10.1515/bc.2008.064

